# The Role of the Immune System in Pediatric Burns: A Systematic Review

**DOI:** 10.3390/jcm11082262

**Published:** 2022-04-18

**Authors:** Tomasz Korzeniowski, Paulina Mertowska, Sebastian Mertowski, Martyna Podgajna, Ewelina Grywalska, Jerzy Strużyna, Kamil Torres

**Affiliations:** 1 Chair and Department of Didactics and Medical Simulation, Medical University of Lublin, 20-093 Lublin, Poland; t.korzeniowski@gmail.com (T.K.); kamil.torres@umlub.pl (K.T.); 2 East Center of Burns Treatment and Reconstructive Surgery, 21-010 Łęczna, Poland; jerzy.struzyna@gmail.com; 3 Department of Experimental Immunology, Medical University of Lublin, 20-093 Lublin, Poland; sebastian.mertowski@umlub.pl (S.M.); 50618@umlub.pl (M.P.); ewelina.grywalska@umlub.pl (E.G.); 4 Chair and Department of Plastic, Reconstructive Surgery and Burn Treatment, Medical University of Lublin, 20-093 Lublin, Poland

**Keywords:** wound healing, burns, immune response, burn shock, immune system

## Abstract

Burns are one of the most common causes of home injuries, characterized by serious damage to the skin and causing the death of affected tissues. In this review, we intended to collect information on the pathophysiological effects of burns in pediatric patients, with particular emphasis on local and systemic responses. A total of 92 articles were included in the review, and the time range of the searched articles was from 2000 to 2021. The occurrence of thermal injuries is a problem that requires special attention in pediatric patients who are still developing. Their exposure to various burns may cause disturbances in the immune response, not only in the area of tissue damage itself but also by disrupting the systemic immune response. The aspect of immunological mechanisms in burns requires further research, and in particular, it is important to focus on younger patients as the existence of subtle differences in wound healing between adults and children may significantly influence the treatment of pediatric patients.

## 1. Introduction

**Figure 1:**
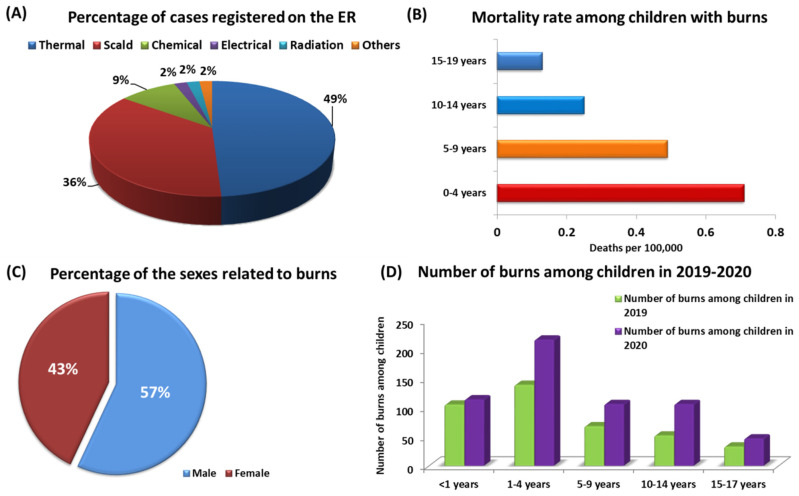
Statistics of burns among children: (**A**) percentage of burns recorded in the ER, including the cause of the injury; (**B**) child mortality rate as a result of burns by age category; (**C**) percentage of burns by patient’s sex; (**D**) number of burns among children in 2019–2020, taking into account age categories based on [16,18,22].

Burns are one of the injuries that damage not only the skin but also deeper tissues. Most often, these injuries are caused by exposure to high temperatures, overexposure to the sun or other radiation, and skin exposure to a chemical agent or electric shock (Figure 1A) [1,2,3]. Burns are mainly characterized by serious damage to the skin, which causes the death of the affected skin cells, which are the body’s first line of defense against harmful environmental factors, injuries and infections [4,5]. The consequences of a high temperature on human skin (depending on the temperature and duration of exposure) may lead not only to local but also systemic damage [6,7,8,9,10]. According to the information presented by the World Health Organization (WHO) and the Center for Disease Prevention and Control (CDC), burns are one of the most common causes of home injuries, and children under 19 are particularly vulnerable [11,12]. As shown in the literature, burns are one of the causes of the loss of Disability Adjusted Life Years (DALYs), mainly in middle- and low-income countries (with a DALY of 96 per 100,000) [13,14]. However, burns are not only a significant cause of increased morbidity and disability among children but also one of the major causes of death in this group of patients. According to WHO data, nearly 180,000 people die from burns each year [11,15]. Although child mortality rates have declined in recent years, this trend only applies to highly developed countries, e.g., in the United States, the death rate of children due to burns fell from 0.71 per 100,000 in 2004 to 0.39 per 100,000 in 2018 [16,17]. Still, the percentage of children dying from burns in low- and middle-income countries is almost 11 times higher than in highly developed countries and amounts to 4.3 deaths per 100,000 [18,19]. Moreover, the available literature data also show that the highest risk group for burns is children aged 0–4 years, among whom the mortality rate of 0.71 per 100,000 deaths was recorded in the United States in 2018. This is 1.45× higher than the group of children aged 5–9 and 2.84× higher than that of children aged 10–14 and also 5.46× higher than of adolescents aged 15–19 (Figure 1B). Undoubtedly, such a discrepancy results from stages of child development in individual age groups and their self-awareness, as children up to the age of 4 begin to move independently on their own and are extremely curious about the world [16,20]. Additionally, medical reports analyzed by the WHO show that male patients are hospitalized much more often as a result of burns than females. These tendencies are confirmed by the collected data, which show that in each age group, men are more likely to get burns, accounting for about 57% of eruptions registered cases (Figure 1C) [16,21]. Retrospective studies conducted by the American Academy of Pediatrics showed that the development of the COVID-19 pandemic also contributed to the increase in the number of burns among children under the age of 19. The presented data show that in 2020, there was a rise in pediatric patients with burns by 48.6% compared to the year 2019 (Figure 1D). The highest increase was observed in the age group 10–14 (by 103.85% compared to 2019) and in the groups of 5–9 years (an increase of 55.88%) and 1–4 years (an increase by 56.11%) (Figure 1D). As researchers point out, this is undoubtedly due to the prolonged stay of children at home as a result of the introduction of remote learning [22].

Children are a special group of patients in whom burns are associated with much greater and more serious consequences than in the case of adults. First, these differences are due to the structure of the skin itself, which is much thinner in children than in adults. This means that both the time and energy required to cause a burn in children is shorter than in adults, resulting in injuries that occur much faster and are also deeper than in adults. It also leads to several local or systemic changes in such patients who are exposed to wound infections, prolonged healing, hypothermia, development of severe inflammatory reactions, hypermetabolic syndrome, and immunosuppression [23,24,25]. Damage to the skin as a result of burns leads to dysregulation, the loss of the protective barrier, but also neurosensory and metabolic functions (disturbances in water homeostasis and thermoregulation), as well as immunological functions are impaired. The immune system plays an extremely important role in the body’s response to burns, determining the prognosis and recovery time for many patients, especially pediatric ones. Tissue damage during a burn causes a strong inflammatory reaction, leading to impaired immune function. Extensive inflammation developing in children up to 19 years of age causes the release of inflammatory mediators and markers, the accumulation of which may cause systemic inflammatory response syndrome (SIRS), which in turn leads to dysregulation of the immune homeostasis of the human body but also to the development of multiple organ dysfunction syndrome (MODS) [26,27,28]. Burn-induced changes affect the functioning of many immunological cells and the compounds they secrete (mainly cytokines), which impair the innate and acquired mechanisms of the patient’s immune system [29]. SIRS developing in the patient’s body contributes to the increase in immunosuppression, which makes the body more susceptible to bacterial infections and the development of sepsis [30]. Additionally, the hyperinflammatory reaction observed in the course of sepsis leads to the formation of the state of “immunoparalysis” in the body, which is particularly dangerous in pediatric patients [31]. The immunoparalysis syndrome can affect both innate and adaptive mechanisms of immune responses, in which reduced HLA-DR expression is observed in monocytes, as well as the reduced ability to produce cytokines by leukocytes, the presence of lymphopenia, and the increased expression of inhibitory immune checkpoints on the cell surface such as PD-1 [32,33,34,35].

The detailed role of the immune system and the specific immune cells and its importance in the evolution of tissue changes in burns is an extremely important research topic aimed at a better understanding of the mechanisms influencing the healing of injuries and restoring the immune balance. The purpose of this review is to gather information on the pathophysiological effects of burns in pediatric patients, with particular emphasis on local and systemic responses. In addition, we would like to present the important role of the immune system in the course of burns and the healing process of this type of injury, which, as a result of dysregulation of the mechanisms of innate and acquired responses, leads to immunosuppression that threatens life and health of children.

## 2. Materials and Methods

### Search Strategy, Study Selection, and Data Extraction

The literature analysis was carried out on the PubMed database where the search for available articles was performed based on the following keywords: “Burns in children”, “Pediatric burns”, “Paediatric burns”, “Immunity”, “Immune response”, “Immune system”. The time range of the searched articles was established for the years 2000 to 2021, and filters related to the type of articles (clinical trials, review, systematic review, book) were used. Repetitions were rejected from the found articles. The suitability for the inclusion of each work into the publication was thoroughly assessed. Eventually, 88 articles were included in the review.

## 3. Assessment of the Effects of Burns in Pediatric Patients

Burns are an extremely dynamic type of damage to the body, the scale of which in the initial stages after contact with the agent is difficult to estimate due to the problems with assessing the depth and extent of the resulting wounds on the patient’s body [36]. The speed of assessing the severity of such an injury (burn size, percentage of burnt the body surface, fluid resuscitation) influences the selection of the appropriate clinical procedure and its subsequent consequences (development of inflammation, hypermetabolic syndrome, tissue infections or risk of death), especially in the case of pediatric patients [37].

The size of burns in children will be different in relation to adults due to changes in the percentage of the patient’s body surface area resulting from their individual development. There are several methods of counting the extent of burns, expressed as a percentage of the total body surface area [19,38]. Typically, adolescents (over 14 years of age) and adults use the “rule of nine”, with each upper limb accounting for 9% and each lower limb accounting for 18% of the total body surface area. Additionally, the head accounts for 9%, the torso for 18%, and the perineum for 1% of the total body surface [39,40].

Contrary to the rule of nines, the Lund and Browder chart is used very often to assess the extent of burns in pediatric patients, which considers the age of the burned person [41]. It characterizes particular regions of the pediatric patient’s body in detail, where the percentage of burns to the head decreases and the percentage of burns to the legs increases with the age of a child, which makes it a much more effective tool for assessing the extent of burns in such patients [42].

### 3.1. Pathophysiological Effects of Burns in Pediatric Patients

The consequences of burns lead to the loss of basic functions of the skin, which is thinner in children than in adults. Additionally, children have an increased metabolism, increased heat loss (conditioned by lower body fat content) and are exposed to increased water loss due to evaporation [43]. The effects of burns in pediatric patients are undoubtedly more dangerous and burdened with a higher risk of complications than in adults. Younger children are at risk of developing hypothermia and increased evaporation loss due to a greater surface area to weight ratio [44]. There is also an increased risk of damage or obstruction of the airways (due to the smaller opening of the airways), possible laryngeal edema, sepsis, hypervolemia or dysfunction of internal organs (heart or kidneys) [45,46,47]. The inflammatory reaction following burns in pediatric patients is also usually stronger than in adults, which is also associated with increased susceptibility to the development of a hypermetabolic state [48]. Due to the fact that children are still in the growth period, the management of wounds or scarring caused by burns is an additional compilation, as the applied treatments must allow the skin to grow and maintain its elasticity in order to adapt to each stage of the patient’s development [24].

All pathophysiological effects and consequences of burns occurring in pediatric patients can be divided into local and systemic reactions [10,49].

#### 3.1.1. Local Response to Burns in Pediatric Patients

Thermal injuries that cause burns occur in the human body in two characteristic stages. First, coagulation-type necrosis develops in the epidermis and tissues. This is an acute type of necrosis that causes degeneration of protein fibers, turning albumin into an opaque, compact structure. Structural proteins are also denatured, which results in the inhibition of proteolytic activity [50]. The next step is cell lysis damage that occurs as a result of the progression of ischemic skin damage (24–48 h), caused by the development of vascular thrombosis [51,52]. Within the cells of the immune system, platelets and leukocytes adhere to the surface of the vascular endothelium, while in the complement system, cytotoxic T lymphocytes (Tc) are activated, and the burn tissue itself develops into a site open to various types of infections [53]. There are also several local lesions within the skin as a result of burns. Three distinctive regions are formed: the coagulation (necrosis) zone, the stagnant (ischemic) zone, and the outermost hyperthermic (inflammatory) zone [54]. In the first zone, structural proteins coagulate, causing irreversible tissue damage. In the second one, tissue perfusion decreases, but the cells present in this area are still alive, and the application of a treatment that increases tissue perfusion may save them. Within the third zone, tissue perfusion is increased, and the blood vessels develop characteristic dilation due to the development of inflammation surrounding the burn. Clinical data show that tissues in this area require 7–10 days to regenerate, which may be prolonged when infection occurs [55,56].

#### 3.1.2. Systemic Response to Burns in Pediatric Patients

**Figure 2:**
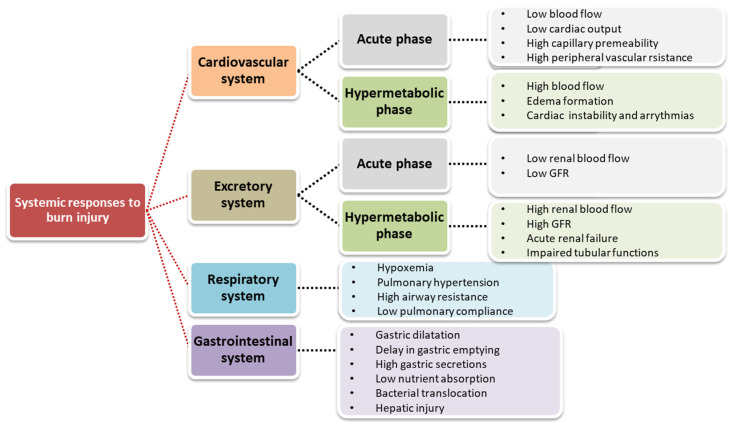
Systemic response to burn injury based on [47].

The systemic response to burns affects almost all internal organs of the patient’s body (e.g., heart, kidney, liver). In severe burns, cytokines and other inflammatory mediators are released in excess in both burn and non-burn areas. These mediators cause narrowing and dilation of blood vessels, an increase in capillary permeability, and the development of edema both at the burn site and in distant organs. Pathological changes also occur in the metabolic, cardiovascular, renal, gastrointestinal and coagulation systems (Figure 2) [47,57,58].

**Figure 3:**
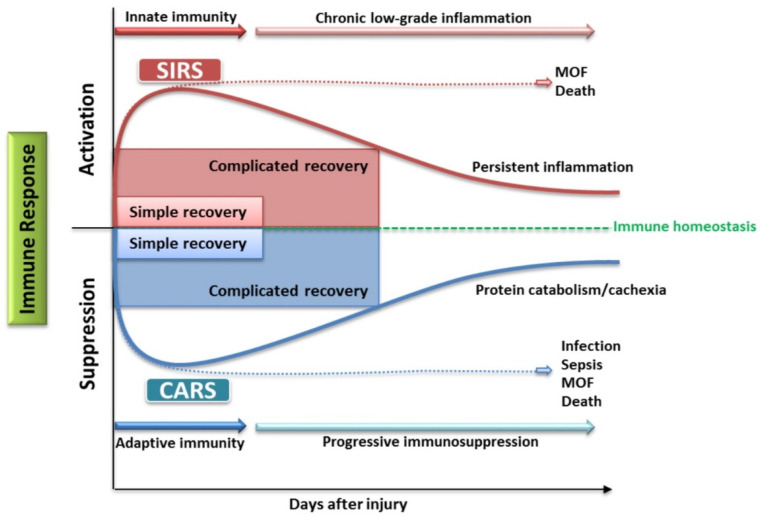
Importance of maintaining immune homeostasis in burns based on [59,60].

The extensive inflammatory reaction of the body to burns can alter the immune response. Here, we can observe the formation of a pro-inflammatory phase that results in the development of SIRS, as well as an anti-inflammatory phase, known as the compensatory anti-inflammatory response syndrome (CARS). Only the balance of these two phases will guarantee therapeutic success. Otherwise, developing SIRS or CARS responses will lead to the development of MODS, infection, sepsis, and even death of the patient (Figure 3).

#### 3.1.3. Burn Shock

When the area of burn of the body exceeds 30%, the body releases kinins from the burn area into the bloodstream (histamine, bradykinin, serotonin) and inflammatory mediators, e.g., cytokines and thromboxanes, prostacyclins, prostaglandins, or leukotrienes, which, when reaching high levels, cause a systemic response [61]. This leads to damage to the endothelium and, consequently, to the displacement of fluids between individual fluid spaces. Plasma finds its way into both tissues damaged by burns and healthy ones. As a result of these processes, the amount of fluid in the vascular bed decreases rapidly, and therefore, burn shock occurs. The burn shock period can be examined in three periods: early, intermediate, and late [62,63].

The first of these, also known as the seepage period, covers the first 36–72 h after the onset of the injury. Then, blood vessels dilate at the burn site, which is accompanied by the release of systemic inflammatory mediators such as histamine, TNF-α, IL-1, IL-6, GM-CSF, INF-γ, or prostaglandins, which are secreted not only from the places of injury themselves but also from healthy tissues adjacent to the burn [64]. The shock generated after the burn is hypovolemic and directly proportional to the extent and severity of the burn. As shown in the literature, in adults, burns of 20% of the body surface area lead to an increased risk of developing hypervolemic shock. In pediatric patients, especially in children under 12, the percentage of body surface burns that correlate with the increased possibility of developing hypervolemic shock is reduced and amounts to 10% [65,66]. Hypovolemia due to circulatory fluid loss caused by edema is observed within the first 2 days and leads to the development of hemodynamic failure due to reduced blood volume [67]. The most common clinical symptoms that indicate the development of hypervolemic shock are pale, moist, and cool skin; tachycardia and hypotension; rapid and shallow breathing; and reduction in urine volume [67,68].

The next period is the intermediate period, also known as the intoxication period, which covers the span of 2 to 4 weeks after the burn occurs [69]. During this time, edema formation ceases, and denatured proteins released from cells enter the circulation, creating a case of intoxication in the body [63]. Approximately 7 days after the injury, the patient’s hemodynamic situation is reversed, accompanied by abnormally high cardiac output and vasodilation [70]. One of the characteristic symptoms of this stage is the appearance of polyuria [71].

The last period of burn shock is the infectious period, in which acute or chronic infections may occur [69]. Both the cellular and humoral immune responses are suppressed at this stage depending on the body surface area that has been damaged by the burn. This leads to the development of lymphopenia, which affects the processes of chemotaxis and phagocytosis [72,73]. Depending on the degree of burn, the activation of T lymphocytes is also weakened, which makes the human body more predisposed/vulnerable to bacterial, viral, or fungal infections [74]. The profiles of cytokines produced by immune cells are also changing, including IL-2, IL-1, IL-6, and IL-8, the concentration of which decreases significantly in the first weeks after the onset of injury, as shown by literature data [63,75,76]. Additionally, increased cell catabolism and the occurrence of capillary leakage reduce the circulating levels of immunoglobulins (IgG, IgA, and IgM) in the peripheral blood [77,78]. Studies by Sobouti et al. show that the decrease in serum immunoglobulin levels is independent of the size of the burn in children. However, they showed that more severe burns in patients were associated with greater reductions in serum levels of IgA, IgM, IgG, and their subclasses [77].

## 4. The Role of the Immune System in Burns

**Figure 4:**
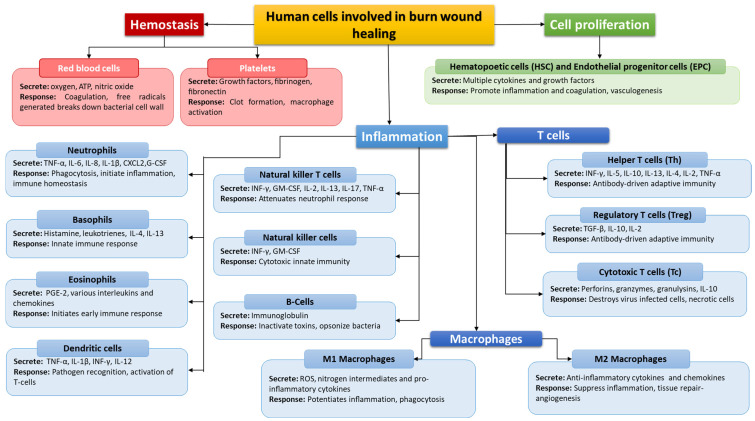
Human cells involved in burn wound healing based on [79,80,81,82].

In burn injuries, the wound healing response is characterized by three stages: inflammation, cell proliferation, and subsequent remodeling. In the first stage, innate immunity is involved, which is triggered by the release of factors such as histamine or cytokines, which cause the expansion of blood vessels, allowing the first line of defense cells such as neutrophils, monocytes, or macrophages to reach the site of damage [79]. The initiated inflammatory process plays an important role in healing burn wounds, as cells such as neutrophils, monocytes, and macrophages can stimulate the activity of fibroblasts and keratinocytes in the proliferation and remodeling phase [79]. Many cells of the immune system are involved in the process of healing wounds resulting from burns, the basic functions of which are presented in Figure 4. All disorders of the immune response at the stage of the healing process of burn wounds may lead to immunosuppression and increased predisposition of patients to various types of infections, which will significantly affect the recovery time [58]. That is why it is so important to establish the mechanisms and functioning of the immune system in patients with burns.

### 4.1. Importance of the Innate Response (Neutrophils, Monocytes, Macrophages, NK Cells)

One of the most important elements of innate immunity in the event of burns is the triggering of a pro-inflammatory cascade caused by thermal trauma. Starting this process may also have significant consequences leading to complications; therefore, determining the participation of individual cells in the process is important [58]. Literature data focusing on the burn area indicate that monocytes, which can transform into macrophages, are important cells involved in the above-mentioned processes. Monocytes and macrophages are involved in immune processes related to phagocytosis (capture and absorption of molecules), including foreign antigens and harmful microorganisms, which include bacteria, viruses and fungi. Macrophages participate in transport of iron to the body tissues, support the production of antibodies and blood vessel formation. Monocytes, although they constitute only a small percentage of all leukocytes, are therefore extremely important for the body [48]. As a result of recognition of molecular patterns, these cells are activated, which in turn leads to the production of chemokines and cytokines [57]. The main mediators secreted by activated monocytes include TNF-α, IL-6 and IL-1β, and IL-10 [57].

The secretion of such compounds influences the mechanism of regulation of the immune and acute phase of response to trauma. In the case of TNF-α, it is involved in the development of a shock-like state associated with thermal damage and sepsis, while IL-1 and IL-6, through their actions, will lead to the activation of granulocytes and the proliferation of T and B lymphocytes [58]. In addition to the above-mentioned mechanisms, activated monocytes, through antigen presentation and expression of, e.g., HLA-DR molecules, can bind to lymphocytes, as a result of which T cells can become activated. In addition, it is indicated that the percentage of monocytes with HLA-DR on their surface is lower in burn patients compared to the control group and is lowest in burn patients who developed sepsis [57]. Monocytes and their subsets (classical subsets of approximately 85 to 95% and intermediate and non-classical subsets of approximately 5 and 9% of all monocytes) play an extremely important role in wound healing. The most important of them seems to be the intermediate subpopulation, which shows high expression of surface markers, such as endothelial growth factors I and II and CXCR4 (C-X-C Motif Chemokine Receptor 4). In addition, as indicated in the literature, this subset after trauma can produce large amounts of proinflammatory cytokines (such as tumor necrosis factor and IL-12), increased levels of which have been detected in severe infections. However, despite extensive research, the role of individual monocyte subsets in burn healing remains unclear [83]. In the case of burns, macrophages play an equally important role as other cells of the immune system and a major role in removing dead cells through phagocytosis. The action of these contributes to stimulating inflammation, but they also secrete compounds that stimulate the healing process of injury, such as fibroblast growth factor, vascular endothelial growth factor, or platelet-derived growth factor [84]. The secretion of the abovementioned factors supports the angiogenesis process. In addition, in the process of wound formation at the stage of proliferation, macrophages produce proteases and stimulate the migration of endothelial cells through the fibroblast growth factor and the production of TGF-β. In addition to supporting the human body in the process of regenerating a burn injury, macrophages also play an important role in defense against pathogens. The performance of so many functions by macrophages is possible thanks to their adaptive function depending on the environment. We can distinguish two types of macrophages: M1 and M2. The first is activated in the classical way; they are used to fight pathogens and are called inflammatory macrophages due to the stimulation of inflammation. In contrast, M2 macrophages are activated by alternative routes depending on the received stimuli from the environment. Due to this ability, they can perform various functions related to the repair of damaged tissues, and they can secrete compounds that reduce inflammation. As a result of thermal damage in wound healing, the phenotype of M1 cells may change to M2 [48,83].

Neutrophils are the next cells involved in the innate immune response. These cells use, e.g., phagolysosomes, free radicals release, or antimicrobial proteases in order to fight pathogens [48]. Neutrophils are also the next cells that migrate the fastest to the burn injury site. However, they show reduced chemotaxis, phagocytosis, and decreased bactericidal capacity, which leads to, despite their relatively large number in burn wounds, impairment of their effector function [48,57]. Reduced neutrophil chemotaxis after a burn can be induced by ceramide-mediated chemotaxis inhibition [84]. As cells of innate immunity, neutrophils can quickly react directly to pathogens by following chemoattractants such as interleukins, chemokines, or bacterial antigens that determine the path that neutrophils are to follow. Weakened neutrophil migration can be supported using antibiotics [57]. Neutrophils, in addition to the function of eliminating pathogens, participate in the process of tissue cleansing. Like monocytes, these cells secrete pro-inflammatory cytokines TNF-α, IL-1β, and IL-6, which indicate the damaged area to other cells, which in turn contributes to the migration of other cells of the immune system to the burned area [48].

Other cells involved in the host’s defense during thermal damage are NK cells (natural killers). These cells are characterized by high cytolytic activity from releasing cytotoxic granules that attach, for example, to infected cells and induce programmed cell death [79]. Rapid response to pathogens (mainly viral) and to abnormal self and infected cells is based on the ability of these cells to kill without recognition of the histocompatibility complex [57]. This type of cell is especially important in burn patients as it enables a rapid response to viral infections that can significantly increase the mortality rate of these patients [57]. NK cells are activated by type I interferon (IFN) and type III interferons. Upon activation, these cells induce the synthesis of type II interferons, IFN-γ and TNF-α [79]. Unfortunately, as a result of thermal damage, despite the unchanged percentage of NK cells, their functioning in the body may be impaired. In the case of pediatric patients, there are no studies available in the literature addressing this aspect, and the limitation of NK cell function has currently only been detected in adult patients. Scientific research shows that a decrease in NK cell activity is observed in burn patients with more than 20% of the total body surface area (TBSA) compared to those with a smaller burn area [57]. The probable mechanism of action of these cells presented in scientific studies is related to the level of IL-2. The lower amount of this interleukin correlates with the lower activity of NK cells. It is also indicated that this correlation may be typical for burn injuries as this type of phenomenon is not detected in people with injuries other than burns [57,58].

### 4.2. Importance of the Acquired Response

Another type of host reaction to burns is the involvement of cells responsible for the acquired immunity, such as lymphocytes. As in the case of innate immune cells, several changes can occur as a result of burns in lymphocytes. One such process may be lymphocyte suppression [57]. In the course of burns, helper T lymphocytes (Th lymphocytes), regulatory T lymphocytes (Treg), and gamma-delta T lymphocytes (γδ) are important subpopulations [57]. According to research data, the skin and epithelial tissues are dominated by a subpopulation of γδ T lymphocytes that express γδ T lymphocyte receptors (γδ TCR) [79]. These cells are also likely to be an important source of chemokines as well as pro-inflammatory cytokines. Additionally, these cells recruit marrow cells to the burn wound to regulate local inflammation [57,79]. T helper lymphocytes can be divided, among others, into subtypes Th1 and Th2. Each type of cell is responsible for a different function: Th1 cells are usually assigned a pro-inflammatory role, while Th2 cells are assigned an anti-inflammatory function. The formation of the Th1 subpopulation is stimulated by IL-12, while the differentiation into Th2 cells is induced by IL-4. As a result of burn damage, the dominant subpopulation in the damaged area is the Th2 type [57]. Another subtype is Treg cells, which are antagonized by Th17 cells. Tregs are regulating cells, including the process of T lymphocyte proliferation, as a result of which, they play an important role in inducing tolerance to transplanted tissue and reducing the inflammatory response. Their action also influences the immune response after burn injuries. According to the literature, these cells are characterized by increased function and occurrence in the lymph nodes after burns. In animal models, it was also observed that the lack of Treg lymphocytes increased the response induced by Th1 lymphocytes [57]. Th17 lymphocytes are a subpopulation of T cells with an antagonistic effect on Treg cells. They act in the recruitment and activation of neutrophils and are responsible for the secretion of IL-17 and IL-22, thus demonstrating a strong pro-inflammatory effect. In children, compared to adults, the level of IL-17 in the early stages after a burn is higher. This may indicate a different IL-17 expression profile compared to adults. This allows for a hypothesis about the existence of differences in the immune system functioning in pediatric patients after burns compared to adult patients; however, this conclusion requires deeper analysis and confirmation in other studies [57].

There is very little information in the literature on the role of B lymphocytes in burns. It is important to mention their lower activity, accompanied by a lower concentration of immunoglobulins in the serum of patients after thermal injuries [57].

Another mechanism that may influence the immune response is co-inhibitory molecules whose task is to regulate the immune response and in particular to suppress this response. Such molecules include the programmed death 1 (PD-1) molecule with its ligands and the CTLA-4 molecule.

The PD-1 molecule is a molecule found on the cell surface. The presence of PD-1 is mainly observed on T lymphocytes and acts as a checkpoint for them as it indicates their condition and state of exhaustion [85]. When the PD-1 receptor binds to its ligands (PD-L1 or PD-L2) on antigen-presenting cells, the pro-inflammatory response is inhibited and results in the process of lymphocyte apoptosis on which this molecule was expressed [57,86]. In the case of burns, an increased expression of the PD-1 molecule has been shown, which may reduce the number of T lymphocytes.

On the other hand, the CTLA-4 molecule is most frequently expressed on T lymphocytes and has an influence on the suppression of the immune response. This molecule belongs to the immunoglobulin subfamily CD28, and its ligands are the receptors CD80 and CD86 on the surface of antigen-presenting cells [87]. Additionally, the CTLA-4 receptor competes with the CD28 molecule to associate with its ligands. As a result of this mechanism and depending on the binding of ligands to this molecule or to its antagonist receptor, the stimulation or inhibition of the immune response of T-lymphocytes may occur. Therefore, CTLA-4 is considered an important factor that allows the maintenance of T-cell homeostasis and their autotolerance to it [87]. As in the case of PD-1 and in relation to the CTLA-4 molecule, it can be viewed as the so-called immune checkpoint. In the case of this receptor, in studies on cancer patients and in patients with viral infections, an improvement in the immune response was achieved after treatment aimed at blocking this molecule. This could indicate that this molecule could also be a potential therapeutic target in the event of burns [57,87].

## 5. Conclusions

The occurrence of thermal injuries is a problem that requires special attention in the case of pediatric patients whose bodies are still developing. Exposing young people to burns (depending on the degree of damage) may cause disturbances in the immune response, not only in the area of tissue damage itself but also in the systemic immune response. Developing severe inflammation caused by thermal trauma may affect the entire body, disrupting the immune homeostasis and affecting the entire process of wound healing and convalescence, as well as the occurrence of severe complications. Therefore, the treatment of burns is complex and requires a holistic approach to the treated patient. Apart from the local treatment of lesions of damaged skin, nutritional and pharmacological support of the whole human body seems to be equally important, as burn patients may show a weaker immune response to infections. To summarize, the aspect of immunological mechanisms in burns requires further research, and in particular, it is important to focus on younger patients. Although the mechanisms of wound healing and responses to burns are similar in adults and children, the existence of subtle differences may significantly influence the treatment of pediatric patients.

## Data Availability

Not applicable.
